# The Comparative Pathology Workbench: Interactive visual analytics for biomedical data

**DOI:** 10.1016/j.jpi.2023.100328

**Published:** 2023-08-09

**Authors:** Michael N. Wicks, Michael Glinka, Bill Hill, Derek Houghton, Mehran Sharghi, Ingrid Ferreira, David Adams, Shahida Din, Irene Papatheodorou, Kathryn Kirkwood, Michael Cheeseman, Albert Burger, Richard A. Baldock, Mark J. Arends

**Affiliations:** a Edinburgh Pathology & Centre for Comparative Pathology, Institute of Genetics & Cancer, University of Edinburgh, Crewe Road, Edinburgh EH4 2XR, UK; b Department of Computer Science, School of Mathematical and Computer Sciences, Heriot-Watt University, Edinburgh, UK; c Experimental Cancer Genetics, Wellcome Sanger Institute, Hinxton, Cambridge, UK; d Edinburgh IBD Unit Western General Hospital, NHS Lothian, Edinburgh, UK; e European Molecular Biology Laboratory - European Bioinformatics Institute (EMBL-EBI), Hinxton, Cambridge, UK; f Pathology Department, Western General Hospital, NHS Lothian, Edinburgh, UK

**Keywords:** Image visualisation, Shared workspace, Image spreadsheet, Visual comparison, Visual analytics, Embedded discussion

## Abstract

Pathologists need to compare histopathological images of normal and diseased tissues between different samples, cases, and species. We have designed an interactive system, termed Comparative Pathology Workbench (CPW), which allows direct and dynamic comparison of images at a variety of magnifications, selected regions of interest, as well as the results of image analysis or other data analyses such as scRNA-seq. This allows pathologists to indicate key diagnostic features, with a mechanism to allow discussion threads amongst expert groups of pathologists and other disciplines. The data and associated discussions can be accessed online from anywhere in the world. The Comparative Pathology Workbench (CPW) is a web-browser-based visual analytics platform providing shared access to an interactive “spreadsheet” style presentation of image and associated analysis data. The CPW provides a grid layout of rows and columns so that images that correspond to matching data can be organised in the form of an image-enabled “spreadsheet”. An individual workbench can be shared with other users with read-only or full edit access as required. In addition, each workbench element or the whole bench itself has an associated discussion thread to allow collaborative analysis and consensual interpretation of the data.

The CPW is a Django-based web-application that hosts the workbench data, manages users, and user-preferences. All image data are hosted by other resource applications such as OMERO or the Digital Slide Archive. Further resources can be added as required. The discussion threads are managed using WordPress and include additional graphical and image data. The CPW has been developed to allow integration of image analysis outputs from systems such as QuPath or ImageJ. All software is open-source and available from a GitHub repository.

## Introduction

**Fig. 1:**
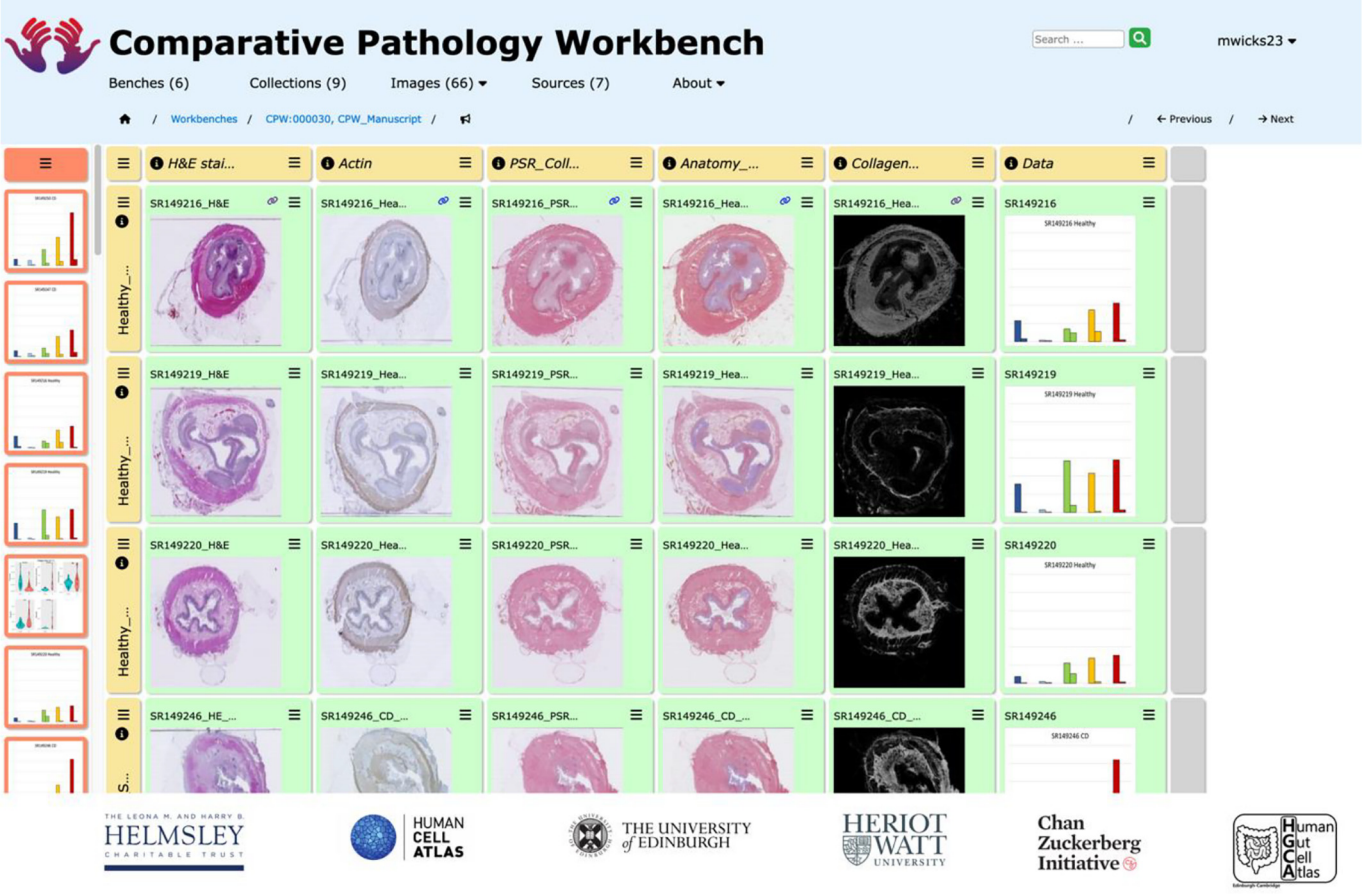
A bench in the Comparative Pathology Workbench system. This shows a typical bench within the CPW system, with the tabular arrangement of rows and columns of cells, and cells populated with images from various sources including graphical presentation of image analysis results.

Pathologists need to be able to compare histopathological images of diseased organs and tissues, for inflammatory and immune disorders, tumours, and many other conditions. Such comparisons may involve large numbers of cases of a specific disease or tumours, to permit comparison of the variation of appearance within human populations. Comparisons are also made between the same or highly similar disease processes in different species, for comparative pathology studies. Currently, pathologists view different cases serially by examining the glass slide sections using a microscope and either retain the diagnostic features in memory while comparing a large series of cases, or take a series of photomicrographs of cases at fixed magnifications and compare these static images. Both of these approaches are limiting, often unsatisfactory and not conducive to easy sharing and discussion of observations. To aid such comparisons in a more dynamic and interactive way, we devised a computer-based approach allowing the pathologist to upload digital images of the scanned histopathological data to be compared within a computerised grid of images, with the images arranged in rows and columns in a way that is meaningful for the study (see Fig. 1). This arrangement allows the user to directly compare images of different cases or different lesions in a variety of subjects or species, matching appropriate magnifications and selected fields of view as required for the study. Requirements gathered from a range of pathologists indicated that this new comparative system should allow regions of interest (ROIs) to be easily marked for further discussion between users. Such mechanisms for discussions coupled with remote access allows for convenient discussion by a group of pathologists at different times or in different places.

Developing and using a visual layout to support the analysis of large-scale data by domain experts has been long researched in the field of *visual analytics.*^1^ Visual analytics emerged from the earlier studies of “sensemaking”,^2^^,^^3^ which is described as a process of initial data capture or “foraging” followed by an expert-driven process of “schema” development to find an optimal representation of the data for the required analysis. Visual analytics was identified as a *Grand Challenge*^4^ to enable and accelerate the analysis of visually presented data (images, spatial and temporal visualisations, graphical layouts, etc.) coupled with real-time updating and high-performance computing. Most of the research effort responding to this challenge has focussed on large-scale and diverse data-types that require novel visualisation techniques and high-performance computing with a number of tools providing “dashboard” style views including automatic updating. Design patterns for visual analytics have been described and systems have emerged using aspects of these patterns for layout and processes, including discussion.^1^^,^^5^

There are a number of commercial applications (e.g. ‘Tableau: Business Intelligence and Analytics Software’)^6^ that incorporate these ideas and various open-access tools such as Google-Sheets, but these are not domain-expert led and tuned to a specific application. In contrast, the underlying design of the Comparative Pathology Workbench (CPW)^28^ has evolved from the sketching of ideas by *domain experts* for an image analytics system useful to pathology research with a particular interest in comparative (cross-species) pathology. The underlying design pattern is a presentation of rows and columns as in a database table or a classical “spreadsheet”, which we term a *workbench* (or *bench* for short). The design criteria include controlled shared access across the internet, simple web-browser-based application with a threaded shared discussion for all data levels including image annotation and markup. The image and genomic/transcriptomic data of interest for this application already have open-access tools for archiving and sophisticated visualisation, so the requirement for this interface was to incorporate these within the workbench in a simple fashion linking out to the full capabilities as required for more detailed visualisation and analysis.

The visual analytics design patterns include a standard row–column schema,^1^ but none of the tools previously available provided the required combination of simplicity with internet wide sharing, annotation, and discussion threads. A spreadsheet design based on Unix tools and systems was developed in 1994 to provide a grid interface for image manipulation,^7^ but this does not appear to have been adopted and adapted to a collaborative working environment. In the next section, we describe the primary purpose and design criteria for the CPW, and the following sections present results with real-world practical applications, and a discussion of the application capabilities.

## Material and methods

### Comparative Pathology Workbench requirements

The practice of clinical and research pathology includes a collaborative process between experts to enable discussions leading to a deeper understanding of the pathological processes involved. For comparative research, this is often a collaboration between internationally distributed groups, making meetings difficult in terms of physical distance and different time-zones. The requirement is therefore a simple interface to organise and view pathology image and other data within an environment that allows easy sharing together with discussion or commentary. It is important that the system is accessible for collaboration on any computer system without the need for special application installation.

In addition to the collaborative working environment, the pathologist-led design process enumerated a number of specific requirements:

1. A simple structured interface that allows comparison of images and analysis charts in 2 dimensions of rows and columns (e.g. image type against experimental sample, or *in situ* hybridisation gene expression stain image against anatomical location).
2. Options to display any visual data (e.g. results of genomic or transcriptomic sequence analysis, or the quantitative image analysis output from a machine learning analysis of histopathology images).
3. Maximum use and re-use of existing systems for image data management and data repositories, such as OMERO,^32^ IDR^31^ or similar image servers.
4. Integration of images under investigation with the visual outputs from analysis tools (e.g. quantitative histopathology analysis using ImageJ, QuPath^8^ or similar software, or transcriptomic analysis with cell-type mapping tools).
5. Lightweight web-based application allowing the user easy and internet-wide access.
6. Sharing model allowing either fully public, or controlled sharing or fully private, as determined by the user.
7. Collaborative tools for capturing discussion threads at each level within the system (e.g. commentary or discussion on individual images, or on images with image annotations or regions of interest, or on a set of images, or at the level of the whole workbench collection of images).

### Implementation

With these considerations, we have developed a 2-D grid style interface analogous to the “spreadsheet” model for numerical data, but specifically for image and other visual data outputs (see Fig. 1). In the first instance, we have focussed on providing integration with 3 key resources. The first is the image management and archiving system *OMERO,*^32^ which has emerged as the lead system for very large-scale repositories of biomedical image data, and is an open-source system for researchers and institutions to set up their own repositories. In particular, OMERO provides the archiving server for the Image Data Resource^9^ currently holding about 335 TB of publicly accessible image data. The second is the open-source histopathology image-analysis system *QuPath*^8^ which delivers very-high quality manual and automated analysis of pathology images, including a range of machine-learning options for training and applying image classifiers for detailed numerical analyses of tissues and cell-types, across the very large images now generated by automated slide scanning. The third is the *Single Cell Expression Atlas (SCEA)*^10^^,^^33^ at the EMBL European Bioinformatics Institute (EBI)^30^ which is an open archive of single-cell RNA sequencing (scRNA-seq) data of direct importance to the research for which CPW has been developed, and represents the type of data that is important to be visualised alongside histological image data.

Although OMERO, QuPath, and SCEA are the initial focus, the CPW system has been designed to allow any open resource with a suitable web-service interface to be incorporated. For example, CPW can operate with the Digital Slide Archive^11^ and the CZI CellXGene system^12^ which are adopted for archiving data within the NIH-funded HuBMAP programme for human single cell analysis.^13^

**Fig. 2:**
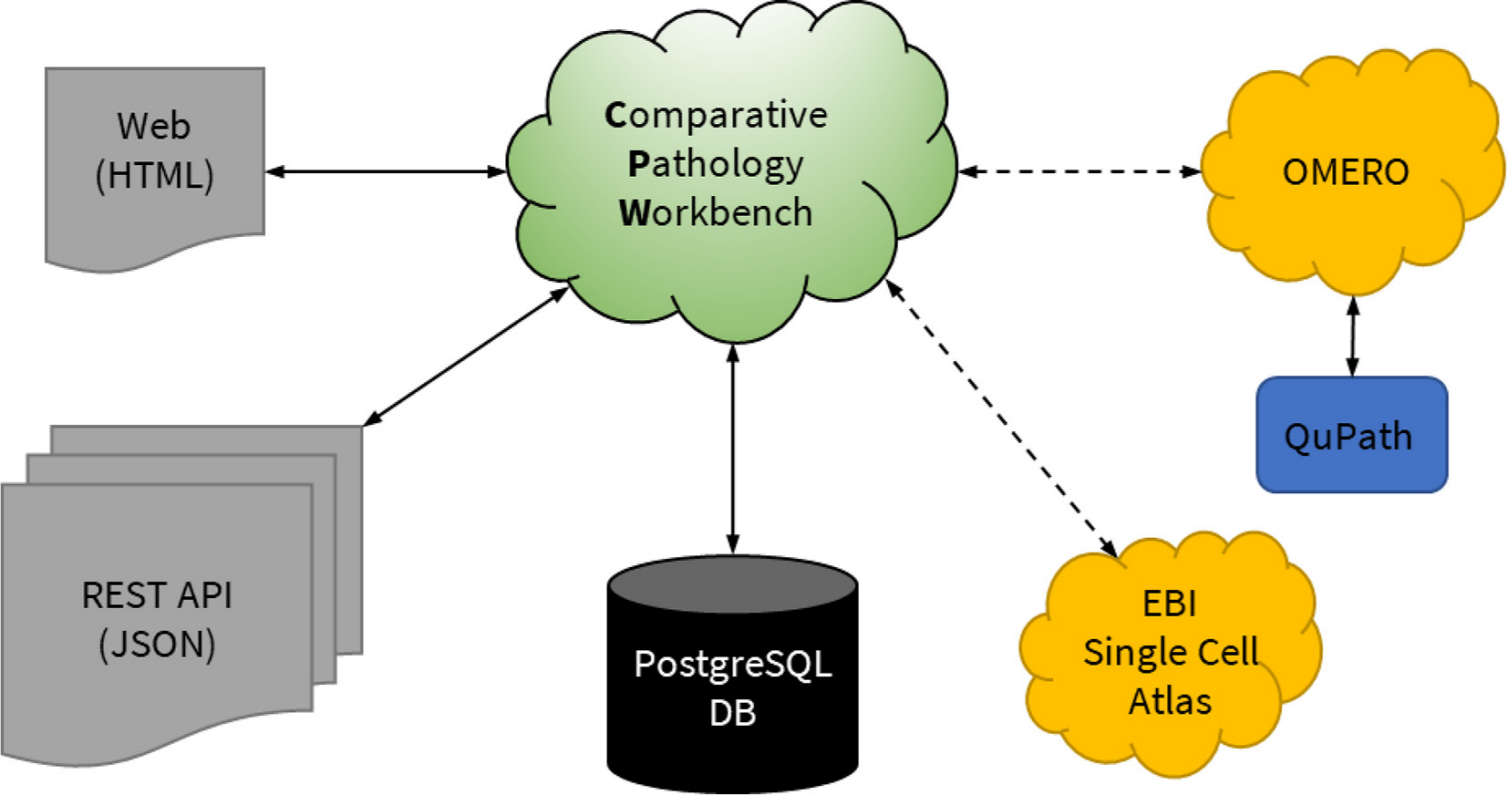
Architectural overview of the CPW software. This overview represents the data flow and exchange in CPW. Minimal data is stored in the CPW, instead only references to supported data and metadata are stored along with the arrangement of the bench as determined by the user. The CPW uses a small PostgreSQL database (DB) to maintain the state of all the benches and collections. It has a comprehensive REST API, which allows for import and export of benches and collections of images. An integration with the metadata held in EBI’s Single Cell Expression Atlas is being developed. This allows for the inclusion of t-SNE or UMAP plots into bench cells. QuPath analysis results can be stored within OMERO, to allow the referencing of these within the CPW. The clouds represent software systems. Normal arrows represent direct connection and ability to manipulate the data within the systems. Dashed arrows represent links or references to other systems.

The CPW has been developed using open-access tools to ensure license-free distribution with the front-end visualisation delivered via a web application written in Python, using the Django web development framework, and incorporating HTML, CSS, and JavaScript to provide the image display, user-interface components and a RESTful API, as well as user and permissions management. The backend SQL database is implemented using Postgres (version: 11) and the discussion threads and management provided use a WordPress instance.^14^ Fig. 2 shows an architectural overview of the CPW software with links through to external services such as OMERO.

## Results

We have implemented the CPW using the “spreadsheet” grid structure to allow the user to arrange data in a tabular format. Each “spreadsheet” instance created by the user is termed a “workbench” or “bench” and each “cell” within a bench can store a URL reference to an image held in OMERO archives or any web-addressable image source. Benches may be shared with other CPW users, for viewing or editing, with a facility provided to allow analysis and discussion between collaborators. In addition, collections of images and other data links can also be created, managed, maintained, and shared with other CPW users.

Minimal original data is stored in the CPW, but derived data from particular analyses and the associated metadata can be stored along with data sharing information and provenance. The CPW uses a Postgres database to maintain the state and associated sharing permissions of all the benches and data collections.

In the context of the CPW we have defined a set of elements:

• **Cell:** An individual component of a bench that can display an image or analysis results.
• **Workbench/Bench:** A user configurable tabular array of cells with the addition of column and row headers that provides an interface for cell editing, copying, and moving using “drag and drop”.
• **Source:** Database archives of image and other data which provide a Uniform Resource Locator (URL) reference to the data.
• **Image:** In the CPW context, any image is linked by an URL plus associated metadata. A thumbnail can be stored locally.
• **Collection:** A named set of images and other URLs which can be shared with other users.
• **Link:** Images and analysis results can be formally linked which can include linking metadata.
• **Commentary:** A discussion thread associated with a cell, row, or column within a bench or the whole bench.

These bench components are described in more detail below followed by descriptions of specific applications of the CPW as a series of exemplars.

### Bench elements and functions

#### Cells

A cell is the basic component of a bench and displays the image reference or analysis content that the user intends for comparison within the bench. The cell holds the link to the original visualisation of that data using the interface provided by the data resource or archive. For example, a thumbnail image of a whole slide high-resolution image of histopathology will link when selected, to the original image displayed in, say, the OMERO image viewer. Each cell holds, in addition to the URL link, a user-defined title, description, and discussion thread link. The cell menu provides a details page to allow editing of all cell content.

#### Benches

**Fig. 3:**
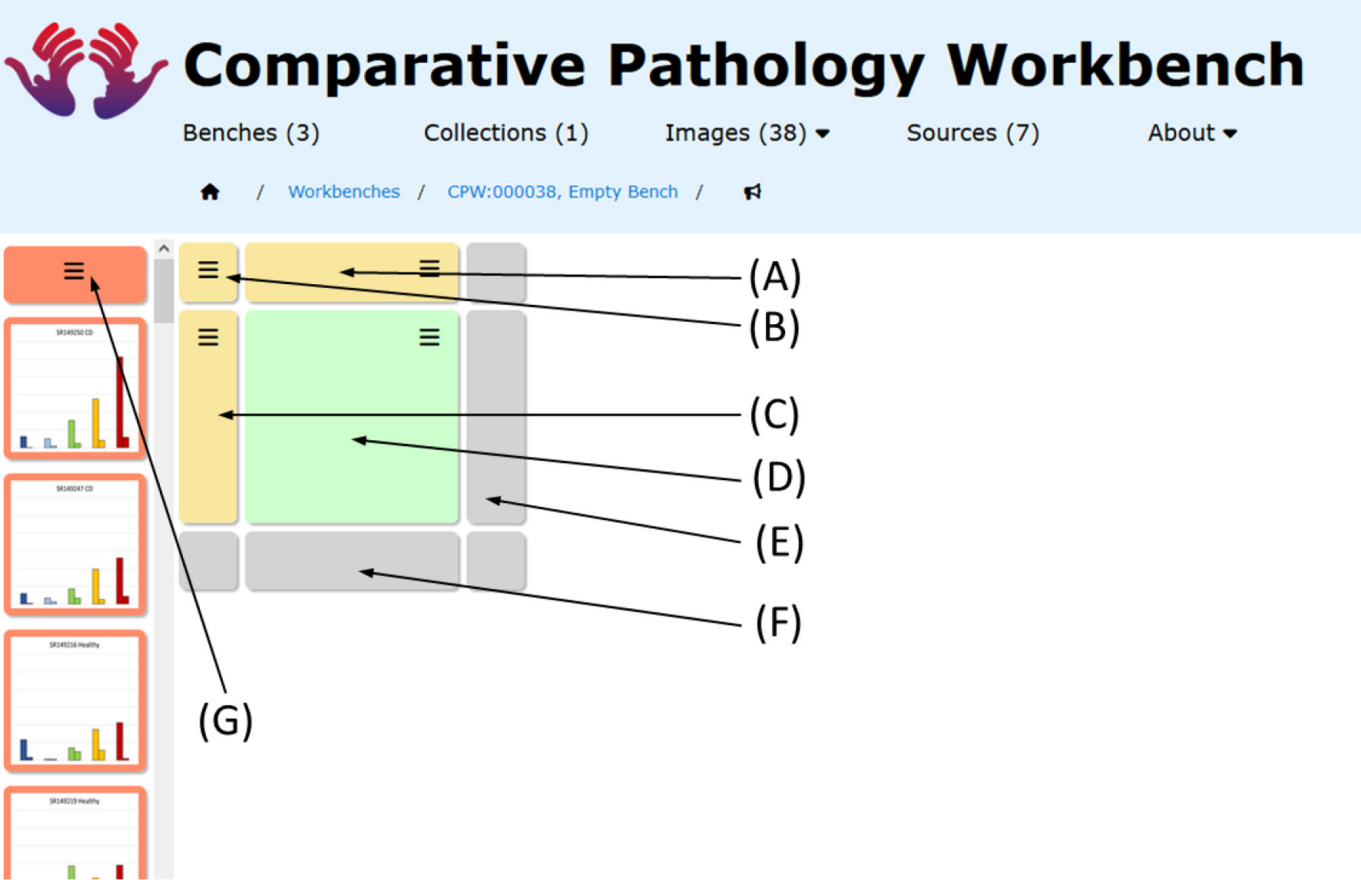
Structure of a cell and bench. The screenshot shows an example bench with all the functions highlighted: (A) Column Header Cell – provides functions to add title to the column for better organisation, move the column around, etc.; (B) Master Cell – provides options to Edit the bench and the commentary associated with it; (C) Row Header Cell – similar to Column Header Cell, provides functions to add title to the whole row for better organisation, move the row around, etc.; (D) Ordinary Cell – this is the cell which gets populated with images or graphs from different sources; (E) Row Footer Cell – a placeholder cells, provides ability to expand the bench by adding more columns; (F) Column Footer Cell – a placeholder cell, provides ability to expand the bench by adding more rows; (G) Collection Cell – cell with the option to pick specific collections to add them to the bench.

A bench is a grid of cells, which can each display a thumbnail of the referenced content (Fig. 3). On bench creation, the user specifies the initial number of rows and columns (up to a maximum of 10 each, with the possibility of adding further rows and columns later), the display dimensions of the cells in the grid and a user-defined bench title and description and each new bench has a unique identifier. The row and column headers can also be given titles and descriptions, as the user requires (Fig. 3: A and C). The number of rows and columns can be changed either by adding or removing individual rows and columns via the column or row menu functions, or by dragging and dropping ordinary cells onto the grey column or row footer cells (Fig. 3: E and F).

The bench page consists of 2 panels: the currently selected image collection is displayed to the left of the page (Fig. 3: G); and the bench cell-array to the right (Fig. 3: B). The image collection provides a “drag and drop” mechanism to populate any give cell with an image.

There are 4 types of cells (Fig. 3) that make up a bench array: *master cell*, *row and column header cells*, *row and column footer cells*, and *ordinary cells*. At the top-left, there is a “master” cell (Fig. 3: B) providing a menu to the main control functions for the bench, including editing the bench detail, bench discussion thread, and bench dimensions. Row and column header cells (Fig. 3: A and C) provide row and column level functions including user-defined labels, title and description. Row and Column footer cells are simply place holder cells, that provide “targets” for the dropping of dragged ordinary cells, resulting in adding extra columns or rows to the bench (Fig. 3: E and F).

An ordinary cell in a bench has a menu offering the functions: amend cell; clear cell; view & edit commentary (Fig. 3: D). The ordinary cell is analogous to the data-cells in a numerical spreadsheet and is the target for displaying selected images or data visualisations.

#### Sources

In the current version, there are 4 types of data source that the CPW can handle:

1. OMERO image archive.
2. WordPress Media Libraries.
3. The EBI Single Cell Expression Atlas (SCEA).
4. Web-based images – a URL plus user-supplied metadata.

OMERO image servers^32^ provide a JSON interface that allows the resources that they manage to be exposed, via unique URLs. The CPW uses this interface to provide a set of screens that enable navigation through the image archive and provides an interface to drill down through the available *groups*, *projects,* and *datasets* stored on an OMERO server. If the datasets are not open to the public, then the user will be prompted to login to the server to allow access. The images within a dataset are then displayed with any defined regions of interest (ROIs) for further processing within the CPW.

The SCEA is a freely available community archive for single-cell RNA sequencing data across all species. Users can query and browse the data and visualise specific analyses such as “t-SNE” and “UMAP” charts derived from selected experiments. Once a specific analysis has been identified the user can “cut and paste” the URL to the CPW, download the generated image of the chart, and upload these to the CPW. These images are then stored locally within the CPW itself for display within a cell as required.

A WordPress^14^ server is included to provide a discussion facility and also as a supplementary mechanism to store miscellaneous images for use associated with a bench. In addition to the discussion threads shown by the CPW, the WordPress server allows an extended “blog” style interface than can be made public for additional input from invited experts or from the community.

#### Images

**Fig. 4:**
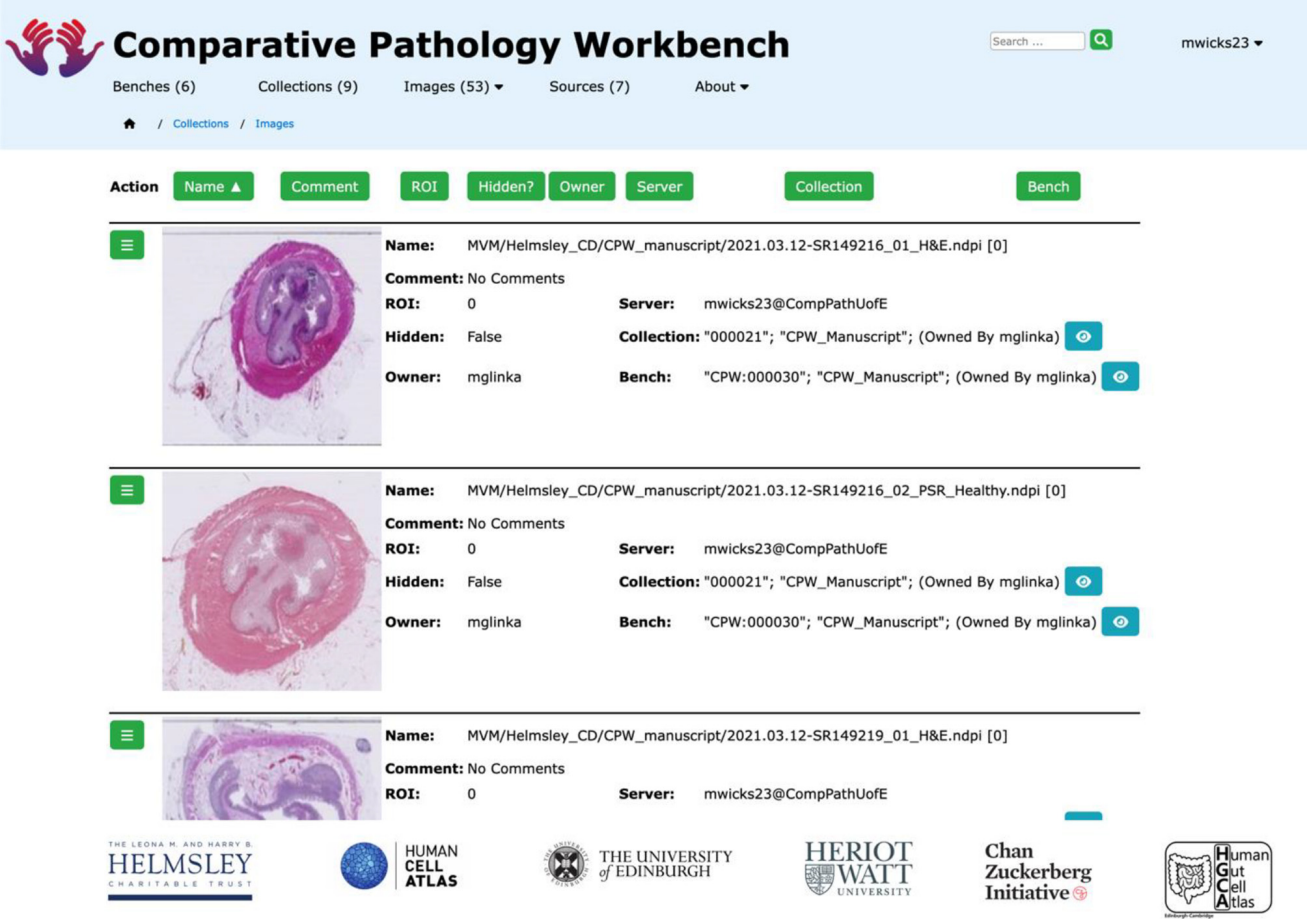
Image collection. A collection of images stored in the CPW, showing the image thumbnail, title, originating server, which collections hold this image, and which Benches display this image; these example images are held on an OMERO server referenced by the CPW.

The CPW does not store any large-scale histopathological images locally within its system; instead, it stores URL references to images that are held on other systems. Images showing analysis outputs often as charts or from web references may be stored locally within the CPW for convenience and to ensure robust access to avoid re-processing. The URL references are to images held on the sources discussed earlier and examples are shown in Fig. 4. In addition to the URL the CPW stores metadata, associated sharing permissions and a user-input name.

The CPW web interface is designed to be viewed in any browser running on any standard computing platform. There are no size limits for images when viewed via the CPW, as the CPW system itself does not store any images, instead storing only references to images stored on another platform, in this case OMERO. Therefore, the image size limitation is determined by the OMERO system which has no theoretical limit – we have tested whole slide images up to 5 GB, but a more typical WSI size is around 1 GB. The CPW server requires a small hardware footprint (e.g. 8 GB RAM, 80 GB hard disc), and this was tested on MacOS (11.7.8), and Linux (Centos 7).

#### Collections

**Fig. 5:**
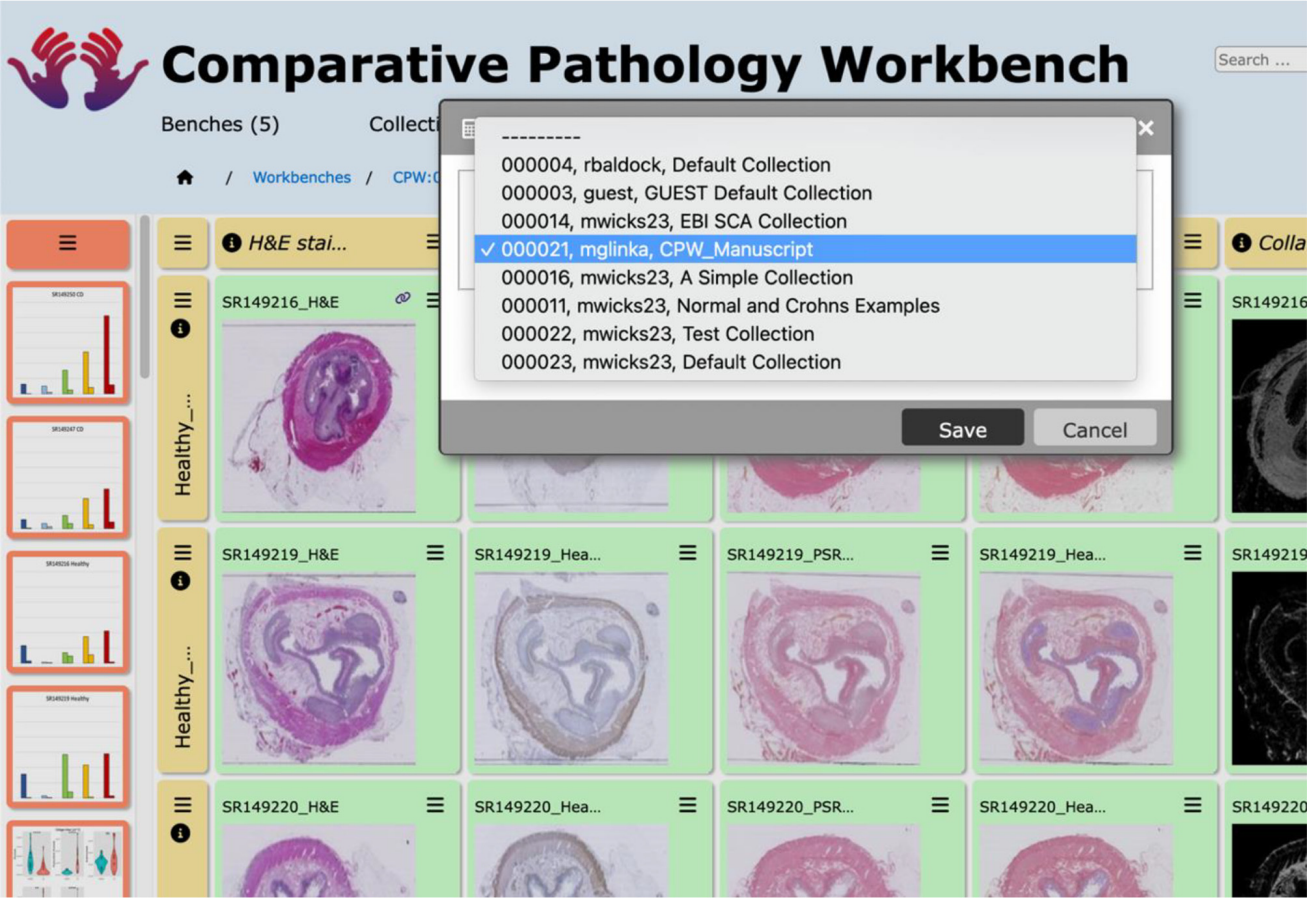
Choosing a collection. As shown previously in Fig. 3: G, a collection can be chosen and the images from the collection moved by Drag & Drop into other cells to occupy them. In fact, the bench can be populated with images and graphs from multiple different collections that can be chosen from the drop-down list, as long as the user has the privileges to view those collections.

A *collection* is a set of images that the CPW user has gathered into a single, named group. When browsing the image sources, the user is given the option to add an image to a collection (see Fig. 4). The images in a collection can then be used by the user to populate individual cells within a bench. Any collection can be shared with editing or viewing privileges with any other CPW user and can be visualised in a browsing interface to check details, metadata, and the image usage within benches and other collections. A collection can be selected for visualisation as a compact list adjacent to a bench to allow images to be copied into individual cells. These interfaces are shown in Fig. 5; collections may consist of images from many different sources.

#### Links

In the CPW, images can be “linked” together, to indicate simple relationships between images. For example, if an image has been generated from another image, using an analysis tool such as QuPath, it is useful to describe such a relationship. The CPW can record simple relationships between pairs of images, along with a comment or a description of the reason for the link. Supporting evidence, for example a detailed description of a segmentation process or a processing script, is provided in a zip file, which must be uploaded at the time the link is created. Whenever an image is displayed in the CPW that has linked images associated with it, a “chain” icon is displayed. Clicking on the chain icon displays the link details, which includes the images linked together, the comment on the link, and the supporting evidence file.

For example, the integration of QuPath to the CPW is indirect, via the linking of 2 images. Images can be created in QuPath and both these derived images and original images from QuPath can be independently uploaded to the CPW and OMERO system and the link between the original and derived images can be defined. This link must have suitable comments and may have a zip file of QuPath outputs (scripts, annotations, masks, or text) attached to the link as well. It is possible to upload single-cell masks and other QuPath or ImageJ masks (or from any other image processing system) derived from image segmentation protocols.

#### Sharing and discussion

In addition to these key CPW components we have built a sharing model to provide users with the capability to share benches and resources with other users of the workbench. Any resource (bench, image, or collection) can be fully private, shared read-only, or shared with full editing permission, although only the owner of a resource can edit the permission status. This sharing within the workbench enables direct collaboration within the set of users registered with a particular instantiation of the CPW. To allow sharing between multiple workbench servers, we have developed and implemented a mechanism to “dump” and “reload” any given workbench, via the RESTful JSON API (see below). This facility is also useful for saving a workbench, for example, as an analysis snapshot associated with a publication, as well as for secure backup.

**Fig. 6:**
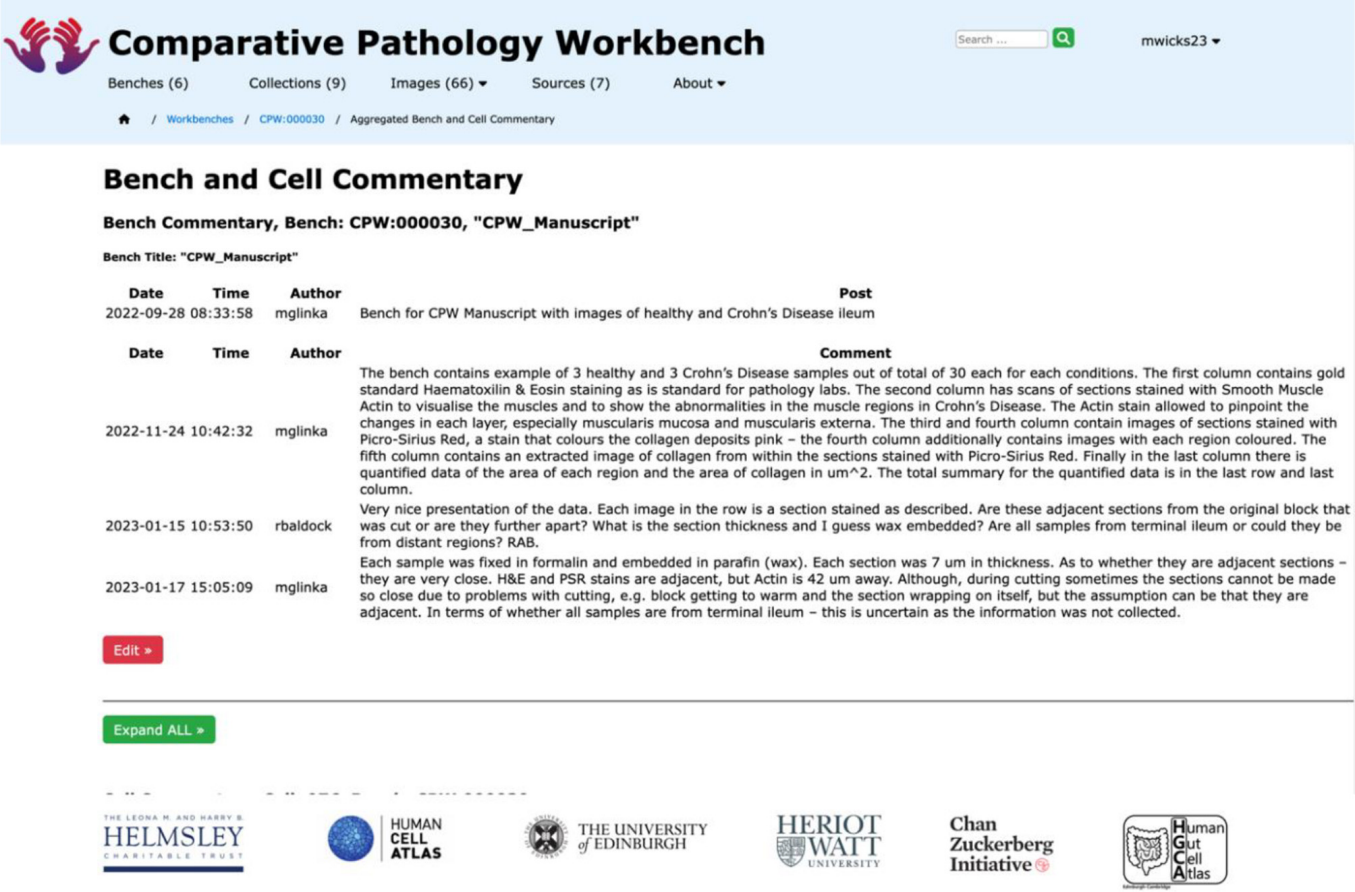
Bench commentary. A screenshot visualising the possible commentary for a bench.

For visual analysis of histopathology images, the research process includes discussion between domain experts, as shown in Fig. 6. For this, we have used WordPress to provide a web-interface to capture the discussion thread or commentary which is analogous to “blog” posts and responses. The WordPress interface can also be used to extend the thread with images and other media for wider sharing. This discussion also serves as a backed-up record of the discussion and decision-making process in the analysis of individual images or sets of images. The discussion can be viewed in the aggregated comments page where both bench and cell comments can be seen and addressed.

#### Search

There are 3 search facilities provided within the CPW: a simple search facility provided in the Header Bar on every page in the system; a Bench search facility on the List Benches page; and a Collection Search facility on the List Collections page.

**Fig. 7:**
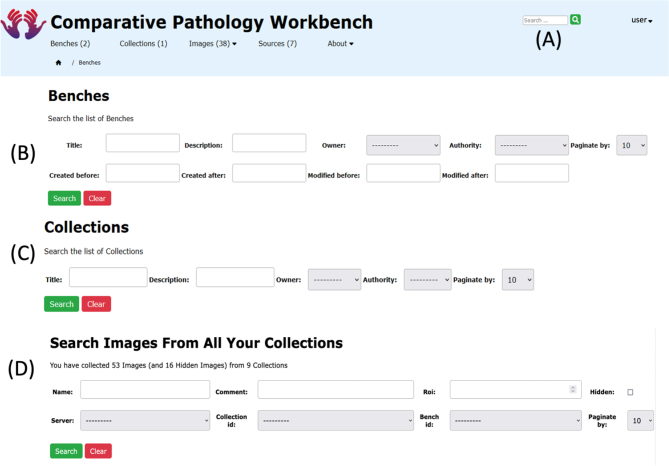
Search functions. A screenshot visualising the search functions: (A) the Simple Search, (B) the Bench Search with the 8 different search criteria, (C) the 4 Collections Search options, and (D) the 7 Image Search options.

Simple Search is a single search box in the System Header Bar, returning a set of Benches whose Title or Description contain this string (Fig. 7: A).

Bench Search allows the user to provide up to 8 different search criteria to find subsets of benches within the system, including the Title, Description, Owner, Authority, Prior to a Creation Date, Post a Creation Date, Prior to a last Modified Date, and Post a Last Modified Date. One, some, or all of these criteria may be supplied by the searcher to narrow the set of returned benches (Fig. 7: B).

Collection Search allows the user to provide up to 4 different search criteria to find subsets of collections within the system, including the Title, Description, Owner, and Authority. One, some or all of these criteria may be supplied to narrow the set of returned collections (Fig. 7: C).

#### RESTful JSON API

The CPW has a comprehensive REST API in addition to the usual web HTML interface. Benches, collections, and images can be manipulated using this API, using the HTTP commands, GET (read), POST (create), PUT (update) and DELETE; PATCH (partial update), and GET to list benches is not supported. This API allows benches, collections, and images to be represented in JSON format (Javascript Object Notation); and this format is used to retrieve, create and update benches, collections and images. The prime motivation behind this interface is to provide a “Dump and Restore” mechanism between instances of the Workbench; and to allow users to copy an existing bench for the purposes of sharing or copying to a different system, should this be required.

### Bench exemplars

Here, we illustrate usage of the CPW via 3 examples of “real-world” applications. Two examples are part of ongoing research programmes and one example is clinically based.

#### DERMATLAS expert diagnosis review

**Fig. 8:**
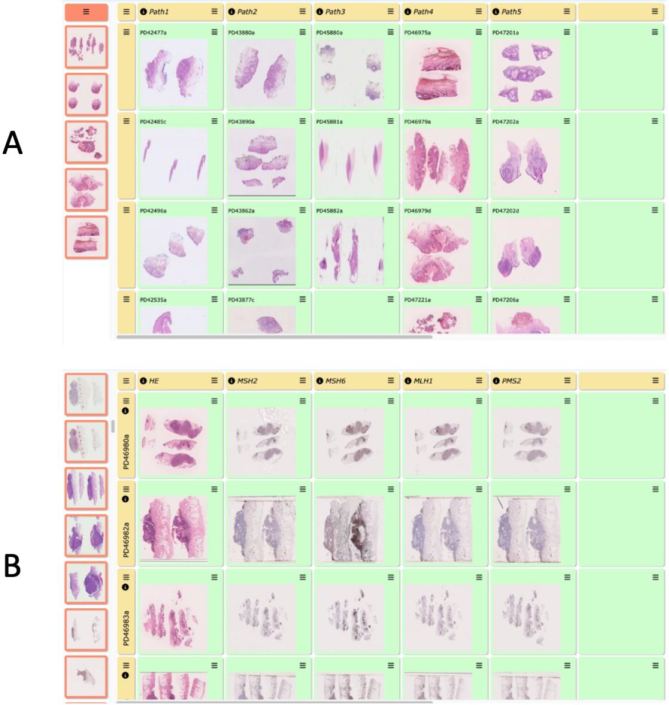
DERMATLAS comparative histopathological analysis workbenches. (A) A typical DERMATLAS workbench for reviewing the diagnoses of a specific skin tumour type. The column headers represent collaborators (“Path1”, “Path2”, etc). Within each column, each cell contains a different skin tumour case (eg. “PD42477a” = anonymised case number, etc). This approach allows all tumours from the same tumour subtype to be compared across the different collaborators for easy review, comparison of key diagnostic features, and discussion of challenging or discrepant cases. (B) A different DERMATLAS workbench for reviewing immunohistochemistry stains performed to aid diagnosis of a particular skin tumour subtype. In this workbench, the column headers represent the different stains used on the samples (eg. HE=Haematoxylin & Eosin, MSH2=immunohistochemical stain for the mismatch repair protein MSH2, etc), with the row headers indicating specific skin tumour anonymised case numbers (e.g. “PD46980a” etc). This approach allows the individual cases to be easily reviewed, compared and discussed by different expert pathologists.

The CPW workbench has been a highly valuable tool for the DERMATLAS project (http://www.dermatlasproject.org/)^29^, which aims to collect 70 types of uncommon and rare skin tumours (including sebaceous neoplasms, sweat gland tumours, other skin appendage tumours, vascular tumours, etc) collected from a number of different countries from around the world (UK, Belgium, France, Canada, USA, Mexico) with collection of around 50 cases for each tumour type (over a thousand scanned slide images have been uploaded to date) (Fig. 8). The CPW has been used to build a series of *workbenches* in order to extensively review this wide range of skin tumours in a practical and pathologist user-friendly way, that is easy to use for any histopathologist.

To be consistent, a workbench has been allocated to each skin tumour type. The grid of rows and columns allowed the user to design the bench in the most efficient and convenient manner for the user (in our case, each column corresponds to a collaborator or source of tumours), as well as direct comparison of skin tumours previously given the same or closely related histopathological diagnoses. As the CPW is accessible from anywhere in the world and from any type of internet-linked computer, all skin tumour diagnoses were reviewed by a panel of 2–4 expert dermatopathologists based in Belgium, UK, and Canada, and specific discussions were possible due to the use of the commentary and annotation functions, from which several diagnoses were modified or changed to new diagnoses. Indeed, using the OMERO viewer, the CPW allows the user to identify and annotate regions of interest (ROIs) for discussion, including making some comments on the scanned slide images in the bench. In addition, OMERO supports a large number of scanned slide formats. Scanned images of immunohistochemistry (IHC) stains were imported to the *workbench* for comparative analysis and discussion by a panel of expert consultant pathology diagnosticians with appropriate expertise in skin tumour IHC interpretation, sometimes as a key component of making the primary diagnosis and at other times IHC was used as a critical molecular pathological test, such as use of mismatch repair IHC for identification of defective mismatch repair in sebaceous skin neoplasms.^15^^,^^16^^,^^17^ Discussions between expert dermatopathologists as part of the DERMATLAS project took place to ensure consistency of histopathological diagnosis and agreement on small regions of tissue identified histologically for targeted high-quality sampling of FFPE (formalin-fixed paraffin-embedded) tumour material and adjacent normal skin FFPE samples for DNA and RNA extraction for comparative tumour genome and transcriptome sequencing studies. This approach provided unique biological and histopathological insights into comparative analyses of the component parts and of the overall diagnoses of a range of uncommon and rare skin tumours, including analysis and expert discussion of the critical diagnostic criteria for diagnosis, molecular pathological testing, and for tumour genomic/transcriptomic analysis.^17^, ^18^, ^19^

##### Crohn’s disease fibrostenosing lesion comparative analysis

**Fig. 9:**
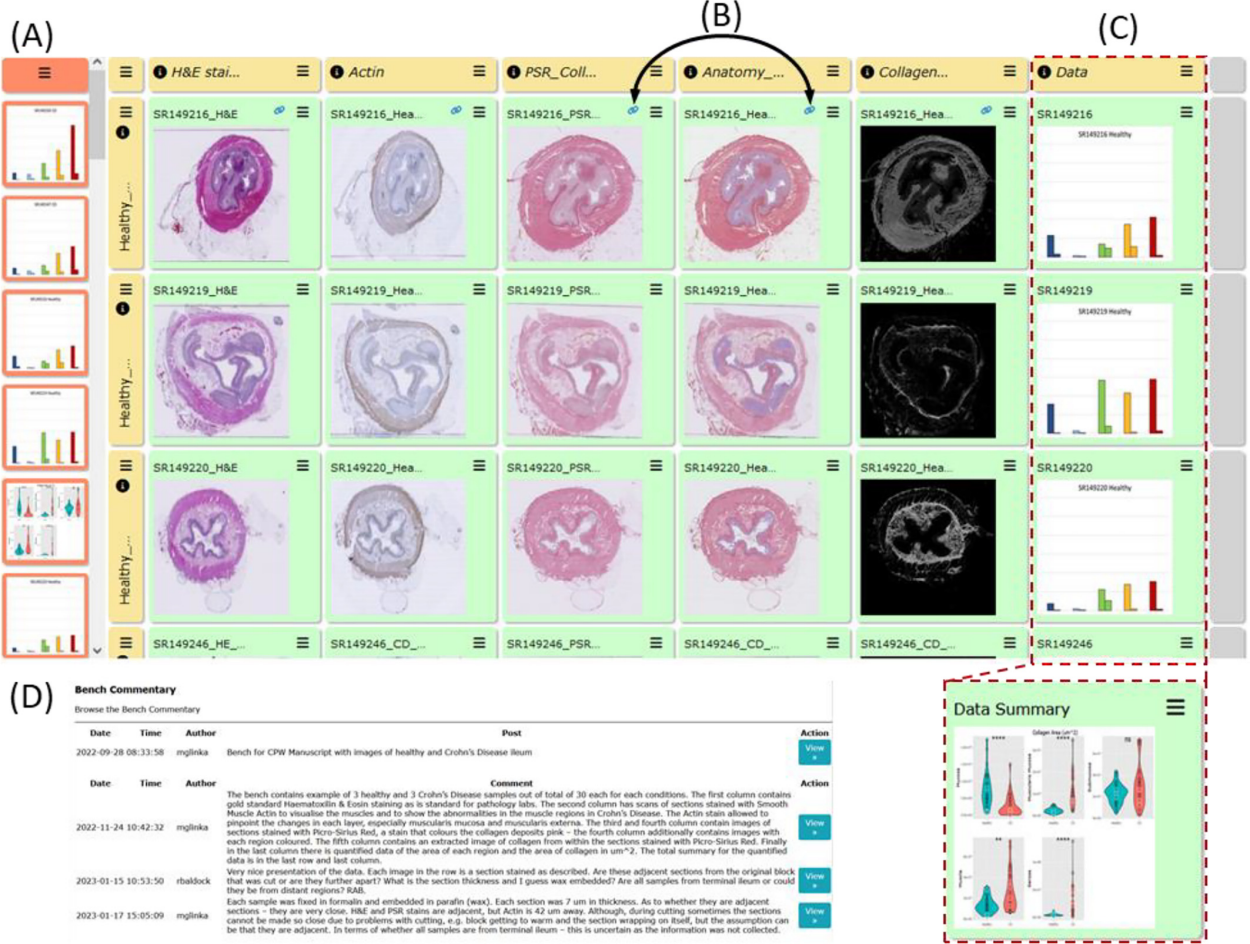
Crohn’s disease fibrostenosing lesion comparative analysis workbench. (A) Visualisation of the workbench displaying images of the histological sections. Rows represent specific samples; columns represent specific stains and/or data related to all samples. (B) Chain link icons representing links that were created between sections to visualise and notify the user that there is a relationship between such sections. (C) Red-dashed line delineated box represents accumulation of all of the data points quantified from annotations. These data represent the surface area size of each region of the ileum as well as the collagen area quantified using QuPath. The summary data can be found in the last row as shown in “Data Summary”. (D) The CPW commentary function allowed for discussion about the workbench images and data to further increase understanding of the integrated morphological, fibrosis-histochemical, and immunohistochemical stains with quantitative evaluation and analysis.

The functionality of the CPW allowed for visualisation of the differences in the morphology of a set of 30 terminal ileal fibrostenosing lesions surgically resected from different Crohn’s disease patients. In addition, a set of 30 normal terminal ileum controls were collected from non-Crohn’s patients (Fig. 5, Fig. 9) collected in the context of the Human Gut Cell Atlas^20^, ^21^, ^22^ and Human Cell Atlas programmes.^13^ Standard histological H&E stained sections, scanned as whole slide images, were uploaded to the bench for these Crohn’s fibrostenotic lesions. Further sections were taken from the same tissue blocks for both histochemical analysis of the amount of collagenous fibrous tissue determined by Picrosirius Red staining and also for a set of immunohistochemical (IHC) stains for a variety of different cell types (B-cells, T-cells, macrophages, smooth muscle cells, etc) for comparison of the histopathological changes between these different cases of Crohn’s Disease fibrostenosing lesions with normal ileum controls.

A shared discussion between researchers and pathologists allowed for identification of additional regions of interest which in turn generated a further focus on in-depth discussions and quantitative analyses of the amount of collagen using QuPath.^8^ The additional regions of interest were then uploaded to OMERO and linked in the CPW (Fig. 9: A and B). The quantified data from QuPath were then uploaded to CPW itself and a summary column was generated (Fig. 9: C), which allowed for better visualisation of the differences between the cases. The functionality of the CPW has allowed images to be shared and provided a discussion platform for experts to gain further insights into the development, locations, and consequences of excessive fibrosis in Crohn’s Disease (Fig. 9: D).

#### Coeliac disease duodenal biopsy histopathology audit

**Fig. 10:**
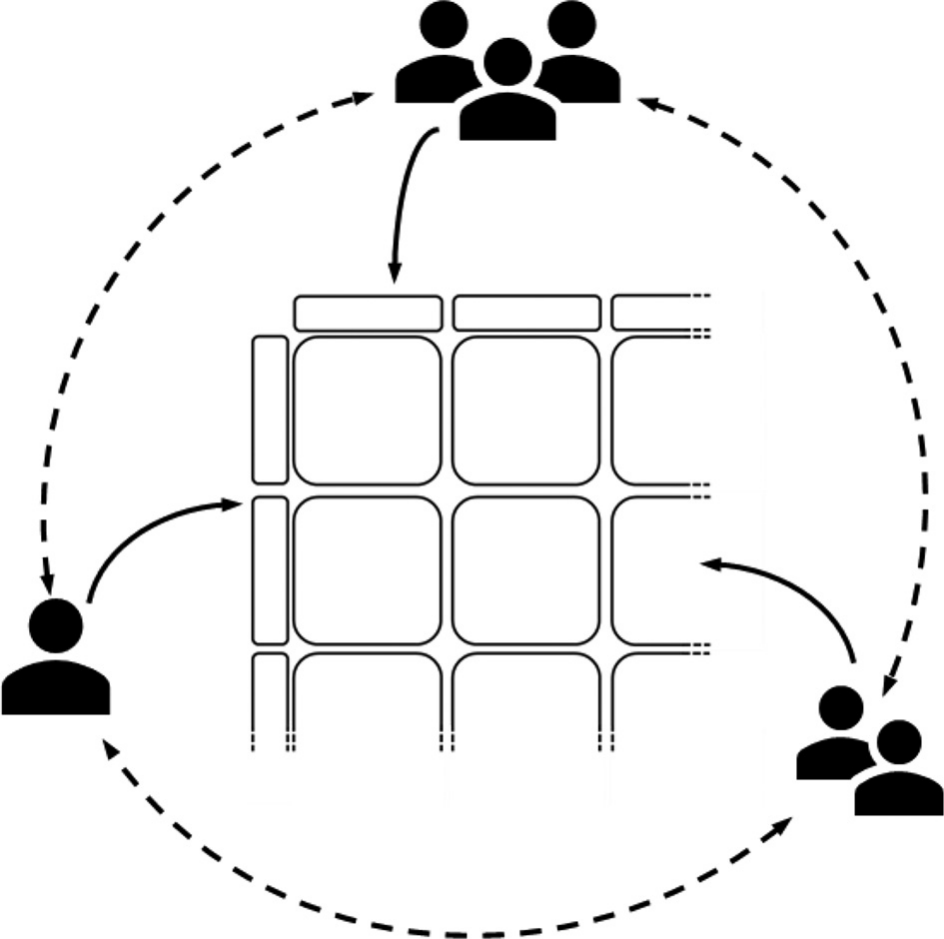
Visualisation of the coeliac disease histopathology audit. The infographic represents how the audit was performed. A number of pathologists simultaneously accessed the workbench from many different locations, edited it, and exchanged their observations (solid arrows), allowing for a rapid exchange of diagnoses and opinions (dashed arrows).

A CPW workbench was set up to use as the platform for a histopathological audit study of a large series of scanned whole slide images of duodenal biopsies showing either normal appearances, or Coeliac disease diagnostic features, including total or subtotal villous atrophy, crypt hyperplasia, and intraepithelial lymphocytosis. A group of 13–17 pathologists including expert gastrointestinal consultant histopathologists, were asked to use the CPW platform to view a series of scanned whole slide images and make a diagnosis of either normal appearances, coeliac disease, or indeterminate/uncertain conclusion.^23^^,^^24^ This allowed an audit study to be rapidly and efficiently performed by this group of pathologists using remote access of the CPW, with the initial round of diagnoses made by the panel of pathologists without knowledge of any other pathologists’ diagnoses or opinions. A small number of discrepant-diagnosis cases were identified that had produced significantly differing diagnoses or opinions amongst the pathologists and the CPW platform was used for review of the histopathological features of these cases by a smaller group of national experts with discussion of their features, using the CPW as a discursive and interactive platform in real-time, aimed at reaching a consensus diagnosis or exclusion of the case from the audit study (Fig. 10).

## Discussion

Having identified a clear need for pathologists to be able to directly compare histopathological images of diseases both between different cases and different species, we designed an interactive system, the CPW, that allows direct and dynamic comparison of scanned whole slide histopathological images at a variety of magnifications. This approach also allowed use of selected regions of interest, to permit pathologists to indicate, and discuss key diagnostic features, with a discursive mechanism, allowing discussions amongst a group of expert and non-expert pathologists and other users, that can be accessed remotely from anywhere in the world. The CPW not only allows comparison of standard H&E stained sections as scanned histopathological images, but images of any other type that can be uploaded to the workbench grid, including histochemical special stains (such as the collagen stain illustrated above), immunohistochemical stains, immunofluorescence stains, *in situ* RNA, or DNA hybridisation stains, macroscopic pathological images, radiological images, and images of molecular pathology investigations, such as single cell transcriptomics or genomics chart images, along with other graphs and charts relevant to molecular analysis of the case. As such, the CPW is very flexible in its use of a wide variety of images. Some of these functions have been illustrated here in the 3 use cases included.

Images can be arranged in rows and columns in a way that is meaningful to the user or group of users involved in analysing the grid of images. For example, multiple images from a single patient’s disease process can be arranged in a column, allowing comparison of multiple patients across the rows in a grid. The user has the flexibility to link selected images as part of an analysis as well as keep notes about such linkage. The selection of regions of interest allows the pathologist or other user to focus on critical diagnostic features of a series of cases, along with writing a commentary about these selected features to promote discussion amongst a group of users invited to join analysis of a particular workbench.

The CPW platform can be accessed from most computers that can run the most frequently used internet browsers (Chrome, Firefox, Safari, etc). Remote access allows pathologists and other users to view the images within a bench at a time of their choosing from anywhere in the world and together with the simplicity of the grid arrangement and the ease of access (once invited with the appropriate weblinks) permits a group of busy expert pathologists to share their expertise in order to confirm diagnoses or to generate new insights following analysis of a range of similar cases in a workbench.

The CPW system also has the potential for use as a histology or histopathology teaching tool, by allowing the teacher or demonstrator to upload a set of histological or other images and directing the students to the appropriate weblink with instructions to view the images, identify particular features (marked by ROIs if necessary), invite students to indicate certain structures by the drawing of their own ROIs (with different coloured ROI lines for several features), provide descriptions, diagnoses, or answers to questions, using the commentary function. This system can also allow real-time online virtual interactions between the teacher and students even if they are not together in the same venue. This potential use as a teaching tool will be explored by us in the coming months, along with follow-up studies on the usability of the CPW by others with collection of responses in answer to questionnaires.

The CPW has some limitations, including the design feature that it works mostly with scanned microscopic whole slide images (stored on OMERO), although it can also display any image type, such as macroscopic pathology images (JPEG, TIFF, or other image formats), amongst others. The CPW has a custom interface to the EBI Single Cell Expression Atlas (SCEA) in order to display SCEA chart outputs (UMAP plots or t-SNE plots), whereas other data sources would require custom interfaces to be constructed and can be developed as required. The CPW can sometimes be slow to render the web page for a large workbench that contains a large number of images (>100) from an OMERO server. We are currently implementing a software upgrade for thumbnail image caching that would speed up web page rendering. The CPW only usefully displays on a desktop or laptop machine and is not suitable with constrained devices with small screens.

The workbench interface has been implemented and currently is in use with a number of pathology research programmes^16^^,^^18^^,^^24^ and an open test version is available from the Centre for Comparative Pathology (CCP) website.^25^ In addition, all software is open-source and available from a GitHub repository.^26^ A detailed user-manual describing the detailed structure and functions of the interface has been written and is also available from both the GitHub repository and the CCP website.

## Conclusions

The Comparative Pathology Workbench (CPW) is a tool designed and developed to enable more rapid and effective pathology analysis via a novel visual interface coupled with sharing and discussion capabilities for widely distributed collaborative teams.^27^ The effectiveness has been demonstrated with 2 research-based exemplars from which further publications are emerging. In addition, a use-case for pathology auditing, in this instance for coeliac disease in duodenal biopsies has been presented to illustrate the effectiveness of the approach. The initial prototype for the CPW was used for student teaching and examination and we envisage significant benefit for this visual analytic design pattern not just in pathology practice, research, and teaching but across many fields that use and compare related sets of images and especially if the analysis involves multiple collaborators contributing different expertise.

The user needs access to the internet to be able to use the CPW and in cases where the network bandwidth is limiting, the loading time for CPW might be prolonged due to the necessity of accessing the image sources.

Future development of the CPW will streamline the existing capabilities and allow interaction with other sources of data, in particular we will bring in the GCA and HCA adopted tools such as the Digital Slide Archive^11^ and CELLxGENE^12^ system and these will be available as an HCA “Analysis Portal” particularly in the context of the Human Gut Cell Atlas.^20^^,^^22^ Other planned developments will be supporting clinical and research pathology in understanding disease progression and comparative analysis across different species for disease modelling.

## Funding

**Chan-Zuckerberg Initiative** Human Cell Atlas grant A-1708-02723, **The Leona M. and Harry B. Helmsley Charitable Trust** entitled “Human Gut Cell Atlas – Normal Intestine and Crohn’s Disease”, grant reference number 1903-03783 and the 10.13039/501100000265**Medical Research Council** grant MR/V000292/1 entitled “The Genomic Atlas of Dermatological Tumours (DERMATLAS)”.

## Ethics approval and consent to participate

Histopathological and radiological image data used for the comparative pathology workbenches was fully anonymised with accordance with the appropriate UK ethical and legal framework. Ethical approval for anonymised tissue section use was obtained from Lothian NRS Bioresource Research Tissue Bank.

## Author contributions

All – manuscript preparation and review; MNW - primary software development and implementation; MG, IF, SD, KK – application testing and review, bench applications; BH, DH, MS – software review and application testing; DA, IP - PI support and data sources; AB – PI support and Comp. Sci. input; MC – original design concepts; RAB, MJA – PI support, original design concepts, applications testing and bench development.

## Declaration of Competing Interest

The authors declare that they have no competing interests.

## Data Availability

All software developed for this project is freely available from the GitHub repository https://github.com/Comparative-Pathology. The exemplar benches and screen snapshots all use version 1.0.0 of the available software as an archive tar- or zip-file from https://github.com/Comparative-Pathology/comparativepathologyworkbench/releases/tag/Release-Candidate-1.0.0.

## References

[1] Heer J., Agrawala M. (2008). Design considerations for collaborative visual analytics. Inf Vis..

[2] Russell D.M., Stefik M.J., Pirolli P., Card S.K. (1993). Proceedings of the INTERACT ’93 and CHI ’93 Conference on Human Factors in Computing Systems.

[3] Pirolli P., Card S. (2005).

[4] Thomas J.J., Cook K.A. (2006). A visual analytics agenda. IEEE Comput Graph Appl..

[5] Zhang L., Stoffel A., Behrisch M. (2012).

[6] Tableau: Business intelligence and analytics software. Tableau. Accessed January 19, 2023. https://www.tableau.com/en-gb/node/62770

[7] Levoy M. (1994). Proceedings of the 21st Annual Conference on Computer Graphics and Interactive Techniques - SIGGRAPH ’94.

[8] Bankhead P., Loughrey M.B., Fernández J.A. (2017). QuPath: open source software for digital pathology image analysis. Sci Rep..

[9] Williams E., Moore J., Li S.W. (2017). Image Data Resource: a bioimage data integration and publication platform. Nat Methods..

[10] Moreno P., Fexova S., George N. (2022). Expression Atlas update: gene and protein expression in multiple species. Nucleic Acids Res..

[11] Gutman D.A., Khalilia M., Lee S. (2017). The digital slide archive: a software platform for management, integration, and analysis of histology for cancer research. Cancer Res..

[12] Chan Zuckerberg CELLxGENE Discover Cellxgene Data Portal. https://cellxgene.cziscience.com/.

[13] Snyder M.P., Lin S., Posgai A. (2019). The human body at cellular resolution: the NIH Human Biomolecular Atlas Program. Nature..

[14] WordPress.com WordPress.com. https://wordpress.com/.

[15] Rashid M., van der Horst M., Mentzel T. (2019). ALPK1 hotspot mutation as a driver of human spiradenoma and spiradenocarcinoma. Nat Commun..

[16] Ferreira I., Wiedemeyer K., Demetter P., Adams D.J., Arends M.J., Brenn T. (2020). Update on the pathology, genetics and somatic landscape of sebaceous tumours. Histopathology..

[17] Ferreira I., Arends M.J., van der Weyden L., Adams D.J., Brenn T. (2022). Primary de-differentiated, trans-differentiated and undifferentiated melanomas: overview of the clinicopathological, immunohistochemical and molecular spectrum. Histopathology..

[18] Ferreira I., Droop A., Edwards O. (2021). The clinicopathologic spectrum and genomic landscape of de-/trans-differentiated melanoma. Mod Pathol..

[19] Wong K., van der Weyden L., Schott C.R. (2019). Cross-species genomic landscape comparison of human mucosal melanoma with canine oral and equine melanoma. Nat Commun..

[20] Burger A., Baldock R.A., Adams D.J. (2023). Towards a clinically-based common coordinate framework for the human gut cell atlas: the gut models. BMC Med Inform Decis Mak..

[21] Helmsley Gut Cell Atlas Roadmap. Helmsley Trust. Accessed February 21, 2023. https://helmsleytrust.org/wp-content/uploads/2023/03/Gut-Cell-Atlas-_-Broad-Roadmap.pdf

[22] Zilbauer M., James K., Kaur M. (2023). A roadmap for the human gut cell atlas. Nat Rev Gastroenterol Hepatol..

[23] Jaeckle F., Denholm J., Schreiber B.A. (2023).

[24] Denholm J., Schreiber B.A., Evans S.C. (2022). Multiple-instance-learning-based detection of coeliac disease in histological whole-slide images. J Pathol Inform..

[25] Centre for Comparative Pathology The University of Edinburgh. https://www.ed.ac.uk/comparative-pathology.

[26] Comparative Pathology Workbench https://github.com/Comparative-Pathology/comparativepathologyworkbench.

[27] McInnes E.F., Meyerholz D.K., Arends M.J. (2023). Concerns about pathology expertise and data quality. J Pathol..

[28] Comparative Pathology Workbench The University of Edinburgh. https://www.ed.ac.uk/comparative-pathology/the-gut-cell-atlas-project/detail/comparative-pathology-workbench.

[29] Dermatlas https://www.dermatlasproject.org/.

[30] Institute EB EMBL-EBI Homepage. https://www.ebi.ac.uk/.

[31] IDR: Image Data Resource https://idr.openmicroscopy.org/.

[32] Allan C., Burel J.M., Moore J. (2012). OMERO: flexible, model-driven data management for experimental biology. Nat Methods..

[33] Single Cell Expression Atlas https://www.ebi.ac.uk/gxa/sc/home.

